# Quantum features of nonlinear coupler with competing nonlinearity

**DOI:** 10.1038/s41598-022-12458-0

**Published:** 2022-05-17

**Authors:** Rafael Julius, Abdel-Baset M. A. Ibrahim, Pankaj Kumar Choudhury, Azrul Nizam Alias, Muhammad Syawal Abd Halim

**Affiliations:** 1 Faculty of Applied Sciences, Universiti Teknologi MARA (UiTM) Perak, Tapah Campus, 35400 Tapah Road, Perak, Malaysia; 2 Faculty of Applied Sciences, Universiti Teknologi MARA (UiTM), 40450 Shah Alam, Selangor Malaysia; 3 Institute of Microengineering and Nanoelectronics, Universiti Kebangsaan Malaysia (UKM), 43600 Bangi, Selangor Malaysia; 4 Faculty of Computer and Mathematical Sciences, Universiti Teknologi MARA (UiTM) Perak, Tapah Campus, 35400 Tapah Road, Perak, Malaysia

**Keywords:** Quantum optics, Nonlinear optics, Quantum simulation

## Abstract

In this work, we examine the quantum features of a multi-waveguide nonlinear coupler exploiting the second-and third-order nonlinearities. The considered system contains four identical channels, each with a single fundamental transverse mode. The essence of this type of nonlinear coupler is to examine the effect of two or more competing nonlinearities on the generated nonclassical features in this class of devices. Here, we consider the case of second harmonic generation, wherein the fundamental harmonic (FH) fields are up-converted in pairs to double-frequency second harmonic (SH) fields, which are then evanescently coupled with the fields from other Kerr nonlinear waveguides. Using the positive P representation of the phase space, the time-evolution of the density matrix could be mapped to the corresponding Fokker–Planck equation of a classical quasiprobability distribution. Using Langevin stochastic equation, an exact representation of the system in phase space led to the demonstration of sub-Poissonian property, squeezing, and entanglement. With more effective squeezing achieved in all channel waveguides, the present system with *χ*^(2)^–*χ*^(3)^ interaction can be a more efficient alternative to other versions of nonlinear couplers such as the quantum optical dimer (QOD) and Kerr nonlinear coupler (KNC). Furthermore, such a structure offers more flexibility in coupled-mode interactions in the form of correlation between the modes in different waveguides. This provides a better mechanism for the generation of enhanced nonclassical effects.

## Introduction

Nonclassical phenomena in quantum optics could be used as resource elements in future integrated optics technologies^1^. Pivoted to this, significant research has been reported on achieving nonclassical effects using coupled oscillators in various implementation designs^2–6^. Among the others, one of the most active systems with the potential to generate a wide range of nonclassical states is the integration of guided wave structures^7–10^. This approach remains advantageous since optical waveguide structures are compatible with photonic circuit applications^11^. Monolithic photonic devices, such as the array of nonlinear waveguides^12^, can generate nonclassical biphoton states through cascaded quantum walks^13^, continuous-variable quantum information processing^14,15^, computation^16^, and quantum state engineering^17^. The advantages of this configuration include the ease with which a potential multichannel system can be developed^18–20^ by eliminating the possibility of distortion due to overlapping of light pulses, and also, providing more stable propagation over long distances, higher transmission speed, and less attenuation compared to its equivalent multimode models^21^. As a source of quantum light, it offers more versatility in coupled-mode interactions. New possibilities of correlation between the modes in different channels are incorporated as a result of adding channel waveguides, and thus, a better mechanism for the generation of nonclassical effects could be established^22–25^.

Waveguiding structure has gained considerable attention in the development of nonlinear phenomena related to the generation of quantum effects^26–28^. We reported before the possibilities of generating enhanced nonclassical states via multichannel interactions exploiting nonlinear waveguides with the second-*χ*^(2)^^22,23^ or third-order *χ*^(3)^^24,25^ nonlinear effects. The basic concept behind this was to enhance the number of interacting modes by increasing the number of *χ*^(2)^ or *χ*^(3)^ waveguides, wherein each system was treated independently. The work remains valuable in terms of quantum communication as a foundation for dense optical networks with high-quality data transfer. Therefore, the potential of expanding the nonclassical effects for *χ*^(2)^–*χ*^(3)^ type interactions must be looked at. Interestingly, varieties of useful physical dynamics would emerge from a system with both *χ*^(2)^ and *χ*^(3)^ nonlinearities^29^. The enhanced nonclassical effects and correlations involving interactions with both *χ*^(2)^ and higher-order nonlinearities have been observed previously, for example in the case of traveling-wave and intracavity second harmonic generation (SHG)^30^, atomic coherence ensemble^31^, asymmetric double quantum wells^32^ and quantum dot^33^. Enhancing nonclassical states such as squeezing and entanglement, in general, could help with quantum communication and information processing.

In the present paper, we aim to investigate a system of multichannel waveguides with opposing *χ*^(2)^ and *χ*^(3)^ nonlinear effects. In this arrangement, a second-order nonlinear waveguide *χ*^(2)^ is positioned in the center, surrounded by third-order nonlinear waveguides *χ*^(3)^. The essence of this type of nonlinear coupler is to examine the effect of two competing nonlinearities on the generated nonclassical features in this class of devices. We consider the case of second harmonic generation (SHG), wherein the fundamental harmonic (FH) fields are up-converted in pairs to double-frequency second harmonic (SH) fields, which are then evanescently coupled with the fields from other Kerr nonlinear waveguides.

An adequate quantum mechanical description of the system could be obtained by constructing the overall Hamiltonian. The time evolution of the system is described via the Von-Neumann equation of motion for the density matrix^34^. By applying the quantum–classical correspondence of positive P representation, the quantum operator equation of the density matrix is converted to a classical Fokker–Planck equation (FPE) of the quasi-probability distribution in phase space^35^. The corresponding Stochastic Differential Equation (SDE) can be derived from the FPE using Ito calculus^36^, and then solved numerically.

We investigate the nonclassical features and correlations by studying the time evolution of photon numbers as well as quadrature variances of the averages of fields over a large number of stochastic trajectories. The paper is organized as—after throwing the introductory remarks in “Introduction” section, we describe the derivation of the equation of motion for the current system in “The equation of motion” section. “Criteria for the nonclassicality” section emphasizes the requirements for nonclassicality, which includes the sub-Poissonian property of the mean photon number, entanglement, and squeezing. “The nonclassical features” section discusses the findings of the investigation toward the possibility of extending nonclassical effects, and “Conclusion” section concludes with a brief overview.

## The equation of motion

**Figure 1:**
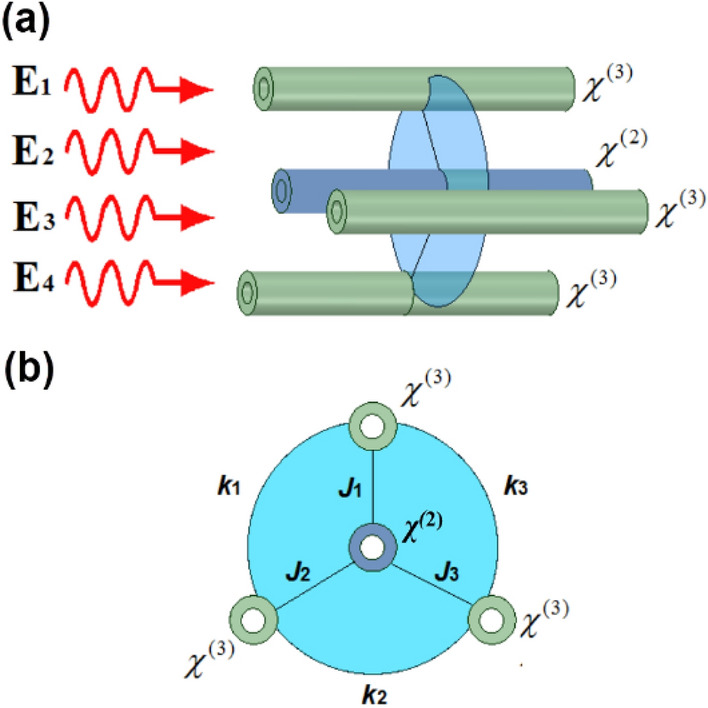
A four-channel *χ*^(2)^–*χ*^(3)^ nonlinear coupler; (**a**) schematic representation, and (**b**) cross-sectional view.

Figure 1 illustrates the schematic of the arrangement of waveguides, wherein we consider a nonlinear waveguide with second-order nonlinearity *χ*^(2)^ at the center, which is encircled (or surrounded) by 03 other waveguides being operated by the third-order nonlinearity *χ*^(3)^ in the vicinity. For generalization of the work, we initially consider the waveguide at the center to be surrounded by an *f*-number of other waveguides, all having the same physical characteristics. Furthermore, each waveguide sustains transverse fundamental mode and is close enough to each other, to allow for evanescent coupling. The total Hamiltonian of the system can be written as

1 $$ \hat{H} = \hslash \left\{ {\hat{H}_{S} + \hat{H}_{N} + \hat{H}_{I} } \right\}, $$

with ℏ being the reduced Planck constant. The Hamiltonian terms $\hat{H}_{S} ,\,\,\hat{H}_{N}$ and $\hat{H}_{I}$ are the pure term that represents the system evolution, the nonlinear interaction term, and the linear coupling term respectively.

In Eq. (1), the first term ${\widehat{{\varvec{H}}}}_{{\varvec{S}}}$ is of the form

2 $$ \hat{H}_{S} = \Omega \left( {\hat{A}^{\dag } \hat{A}} \right) + 2\Omega \left( {\hat{B}^{\dag } \hat{B}} \right) + \omega \sum\limits_{n = 1}^{f} {\left( {\hat{a}_{n}^{\dag } \hat{a}_{n} } \right)} , $$

represents the general evolution of the system in a rotating frame, in which a FH field at frequency Ω generates a SH field at frequency 2Ω in the *χ*^(2)^ waveguide. In the *χ*^(3)^ waveguides, fields are operating at a common frequency *ω*. The bosonic ladder operators $$\hat{A}^{\dag } \hat{A},$$$$\hat{B}^{\dag } \hat{B}$$ and $$\hat{a}_{n}^{\dag } \hat{a}_{n}$$ (with $$n \in \left\{ {1\;to\;f} \right\}$$) satisfy the standard commutation relation [$$\hat{A}_{i} ,\hat{A}_{j}^{\dag }$$] $$= \delta_{ij}$$, [$$\hat{B}_{i} ,\hat{B}_{j}^{\dag }$$] $$= \delta_{ij}$$, and [$$\hat{a}_{i} ,\hat{a}_{j}^{\dag }$$] $$= \delta_{ij}$$ for the FH, SH, and *χ*^(3)^ fields respectively. The second term $${\widehat{{\varvec{H}}}}_{{\varvec{N}}}$$ in Eq. (1) refers to the nonlinear Hamiltonian, and can be written as

3 $$ \hat{H}_{N} = i\frac{\chi }{2}\sum\limits_{n = 1}^{f} {\left( {\hat{A}^{\dag 2} \hat{B} - \hat{A}^{2} \hat{B}^{\dag } } \right)} + g\sum\limits_{n = 1}^{f} {\left( {\hat{a}_{n}^{\dag 2} \hat{a}_{n}^{2} } \right)} , $$

where the strength of anharmonic coupling due to the *χ*^(2)^ and *χ*^(3)^ nonlinear processes in the interaction media is defined by the parameters *χ* and *g*. If *g* is non-zero, the system describes a *χ*^(2)^–*χ*^(3)^ type interaction, whereas setting *g* = 0 essentially eliminates the nonlinear effects in the surrounding waveguides, and the system is reduced to *χ*^(2)^–*χ*^(1)^ type interaction. Here, *χ*^(1)^ refers to the linear susceptibility polarization of light. The third term ${\widehat{H}}_{I}$ in Eq. (1) assumes the form

4 $$ \hat{H}_{I} = \sum\limits_{n = 1}^{f} {J_{n} \left( {\hat{A}_{1} \hat{a}_{n}^{\dag } + \hat{a}_{1} \hat{A}_{n}^{\dag } } \right)} + \sum\limits_{n = 2}^{f} {k_{n - 1} \left( {\hat{a}_{1} \hat{a}_{n}^{\dag } + \hat{a}_{n} \hat{a}_{1}^{\dag } } \right)} + \sum\limits_{n = 3}^{f} {k_{n} \left( {\hat{a}_{2} \hat{a}_{n}^{\dag } + \hat{a}_{n} \hat{a}_{2}^{\dag } } \right)} , $$

describes the evanescent coupling. Herein, *J*_*n*_ refers to the coupling strength between *χ*^(2)^ and *χ*^(3)^, *χ*^(1)^ waveguides, whereas *k*_*n*_ refers to the nearest-neighbor evanescent coupling among the *χ*^(3)^, *χ*^(1)^ waveguides for *f* > 1.

For the Hamiltonian Eq. (1), the time evolution of the density operator can be conveniently defined semi-analytically by the Von-Neumann equation^34^

5 $$ \partial \hat{\rho }/\partial t = - i\hbar [\hat{H},\hat{\rho }]. $$

Using the generic quantum–classical correspondences in positive P representation^35^, the FPE can be derived from Eq. (5). In the deriving process of FPE, it is important to note that different representations use different operator ordering. In the Positive *P* representation, there is no appearance of higher-order derivatives above the second-order, and can thus be precisely mapped into FPE. However, the resulting phase space equation is proportional to the number of transverse modes. A greater number of modes (or waveguides) in the present case leads to a greater number of system equations, especially in channel *χ*^(2)^ due to the generation of SH frequency. Therefore, we limit the system to quad-channel interaction. Recalling the general form of FPE,

6 $$ \frac{\partial P}{{\partial t}} = \left[ { - \sum\limits_{i} {\frac{\partial }{{\partial x_{i} }}A_{i} \left( x \right)} + \frac{1}{2}\sum\limits_{ij} {\frac{\partial }{{\partial x_{i} }}} \frac{\partial }{{\partial x_{j} }}B_{ij} \left( x \right)} \right]P $$

where *A*_*i*_(*x*) and *D*_ij_(*x*) describe the drift and diffusion coefficient respectively. In the present consideration, for *f* = 3, The FPE (6) assumes the following form;

7 $$ \begin{aligned} \frac{\partial P}{{\partial t}} & = \left\{ {\frac{\partial }{{\partial \varsigma_{1} }}\left( {i\Omega \varsigma_{1} - \chi \varsigma_{1}^{*} \xi_{1} + i\left( {J_{1} \alpha_{1} + J_{2} \alpha_{2} + J_{3} \alpha_{3} } \right)} \right) + \frac{\partial }{{\partial \varsigma_{1}^{*} }}\left( { - i\Omega \varsigma_{1}^{*} - \chi \xi_{1}^{*} \varsigma_{1} - i\left( {J_{1} \alpha_{1}^{*} + J_{2} \alpha_{2}^{*} + J_{3} \alpha_{3}^{*} } \right)} \right)} \right. \\ & \quad + \frac{\partial }{{\partial \xi_{1} }}\left( {2i\Omega \xi + \frac{1}{2}\chi \varsigma_{1}^{2} } \right) + \frac{\partial }{{\partial \xi_{1}^{*} }}\left( { - 2i\Omega \xi_{1}^{*} + \frac{1}{2}\chi \varsigma_{1}^{*2} } \right) + \frac{\partial }{{\partial \alpha_{1} }}\left( {i\omega \alpha_{1} + 2ig\alpha_{1}^{*} \alpha_{1}^{2} + iJ_{1} \varsigma_{1} + ik\left( {\alpha_{2} + \alpha_{3} } \right)} \right) \\ & \quad + \frac{\partial }{{\partial \alpha_{2} }}\left( {i\omega \alpha_{2} + 2ig\alpha_{2}^{*} \alpha_{2}^{2} + iJ_{2} \varsigma_{1} + ik\left( {\alpha_{1} + \alpha_{3} } \right)} \right) + \frac{\partial }{{\partial \alpha_{3} }}\left( {i\omega \alpha_{3} + 2ig\alpha_{3}^{*} \alpha_{3}^{2} + iJ_{3} \varsigma_{1} + ik\left( {\alpha_{1} + \alpha_{2} } \right)} \right) \\ & \quad - \frac{\partial }{{\partial \alpha_{1}^{*} }}\left( {i\omega \alpha_{1}^{*} + 2ig\alpha_{1} \alpha_{1}^{*2} + iJ_{1} \varsigma_{1}^{*} + k\left( {\alpha_{2}^{*} + \alpha_{3}^{*} } \right)} \right) - \frac{\partial }{{\partial \alpha_{2}^{*} }}\left( {i\omega \alpha_{2}^{*} + 2ig\alpha_{2} \alpha_{2}^{*2} + iJ_{2} \varsigma_{1}^{*} + k\left( {\alpha_{1}^{*} + \alpha_{3}^{*} } \right)} \right) \\ & \quad - \frac{\partial }{{\partial \alpha_{3}^{*} }}\left( {i\omega \alpha_{3}^{*} + 2ig\alpha_{3} \alpha_{3}^{*2} + iJ_{3} \varsigma_{1}^{*} + k\left( {\alpha_{1}^{*} + \alpha_{2}^{*} } \right)} \right) + \frac{1}{2}\left[ {\frac{\partial }{{\partial \varsigma_{1}^{2} }}\left( {\chi \xi_{1} } \right) + \frac{\partial }{{\partial \varsigma_{1}^{*2} }}\left( {\chi \xi_{1}^{*} } \right)\frac{{\partial^{2} }}{{\partial \alpha_{1}^{2} }}\left( { - 2ig\alpha_{1}^{2} } \right)} \right.\,\,\,\, \\ & \quad \left. {\left. { + \frac{{\partial^{2} }}{{\partial \alpha_{1}^{*2} }}\left( {2ig\alpha_{1}^{*2} } \right) + \frac{{\partial^{2} }}{{\partial \alpha_{2}^{2} }}\left( { - 2ig\alpha_{2}^{2} } \right) + \frac{{\partial^{2} }}{{\partial \alpha_{2}^{*2} }}\left( {2ig\alpha_{2}^{*2} } \right) + \frac{{\partial^{2} }}{{\partial \alpha_{3}^{2} }}\left( { - 2ig\alpha_{3}^{2} } \right) + \frac{{\partial^{2} }}{{\partial \alpha_{3}^{*2} }}\left( {2ig\alpha_{3}^{*2} } \right)} \right]} \right\}P \\ \end{aligned} $$

In terms of a blocked ordered matrix, the diffusion terms of FPE from Eq. (7) may be written as

8 $$ D_{F} = \left[ {\begin{array}{*{20}c} H & I \\ J & K \\ \end{array} } \right], $$

where *I* and *J* are 4 × 4 matrices with zero monomers, while *H* and *K* matrices are defined as

9a $$ H = diag\left[ {\chi \xi_{1} \quad \chi \xi_{1}^{*} \quad - 2ig\alpha_{1}^{2} \quad 2ig\alpha_{1}^{*2} } \right], $$

9b $$ K = diag\left[ { - 2ig\alpha_{2}^{2} \quad 2ig\alpha_{2}^{*2} \quad - 2ig\alpha_{3}^{2} \quad 2ig\alpha_{3}^{*2} } \right]. $$

Equation (9) has the characteristic of diagonal diffusion terms, and therefore, allows for a particularly straightforward factorization of the diffusion matrix, thereby yielding the following set of Stochastic partial differential equations

10a $$ \dot{\varsigma }_{1} = - i\Omega \varsigma_{1} + \chi \varsigma_{1}^{*} \xi_{1} - i\left( {J_{1} \alpha_{1} + J_{2} \alpha_{2} + J_{3} \alpha_{3} } \right) + \sqrt {\chi \xi_{1} } \eta_{1} \left( z \right) $$

10b $$ \dot{\varsigma }_{1}^{*} = i\Omega \varsigma_{1}^{*} + \chi \xi_{1}^{*} \varsigma_{1} + i\left( {J_{1} \alpha_{1}^{*} + J_{2} \alpha_{2}^{*} + J_{3} \alpha_{3}^{*} } \right) + \sqrt {\chi \xi_{1}^{*} } \eta_{2} \left( z \right) $$

10c $$ \dot{\xi }_{1} = - 2i\Omega \xi_{1} - \lambda_{c} \chi \varsigma_{1}^{2} $$

10d $$ \dot{\xi }_{1}^{*} = 2i\Omega_{B} \xi_{1}^{*} - \lambda_{c} \chi \varsigma_{1}^{*2} $$

10e $$ \dot{\alpha }_{1} = - i\left( {\omega \alpha_{1} + 2g\alpha_{1}^{*} \alpha_{1}^{2} + iJ_{1} \varsigma_{1} + ik\left( {\alpha_{2} + \alpha_{3} } \right)} \right) + \sqrt { - 2ig} \alpha_{1} \eta_{3} \left( z \right) $$

10f $$ - \dot{\alpha }_{1}^{*} = - i\left( {\omega \alpha_{1}^{*} + 2g\alpha_{1} \alpha_{1}^{*2} + iJ_{1} \varsigma_{1}^{*} + k\left( {\alpha_{2}^{*} + \alpha_{3}^{*} } \right)} \right) - \sqrt {2ig} \alpha_{1}^{*} \eta_{4} \left( z \right) $$

10g $$ \dot{\alpha }_{2} = - i\left( {\omega \alpha_{2} + 2g\alpha_{2}^{*} \alpha_{2}^{2} + iJ_{2} \varsigma_{1} + ik\left( {\alpha_{1} + \alpha_{3} } \right)} \right) + \sqrt { - 2ig} \alpha_{2} \eta_{5} \left( z \right) $$

10h $$ - \dot{\alpha }_{2}^{*} = - i\left( {\omega \alpha_{2}^{*} + 2g\alpha_{2} \alpha_{2}^{*2} + iJ_{2} \varsigma_{1}^{*} + k\left( {\alpha_{1}^{*} + \alpha_{3}^{*} } \right)} \right) - \sqrt {2ig} \alpha_{2}^{*} \eta_{6} \left( z \right) $$

10i $$ \dot{\alpha }_{3} = - i\left( {\omega \alpha_{3} + 2g\alpha_{3}^{*} \alpha_{3}^{2} + iJ_{3} \varsigma_{1} + ik\left( {\alpha_{1} + \alpha_{2} } \right)} \right) + \sqrt { - 2ig} \alpha_{3} \eta_{7} \left( z \right) $$

10j $$ - \dot{\alpha }_{3}^{*} = - i\left( {\omega \alpha_{3}^{*} + 2g\alpha_{3} \alpha_{3}^{*2} + iJ_{3} \varsigma_{1}^{*} + k\left( {\alpha_{1}^{*} + \alpha_{2}^{*} } \right)} \right) - \sqrt {2ig} \alpha_{3}^{*} \eta_{8} \left( z \right) $$

Herein, the over dot represents the system derivation in the direction of *z*, whereas $\left\{ {\varsigma ,\varsigma^{*} } \right\},\;\left\{ {\xi ,\xi^{*} } \right\}$ and $\left\{ {\alpha_{n} ,\alpha_{n}^{*} } \right\}$ (with $n\in \left\{1,2,3\right\}$) are independent stochastic fields corresponding to the operators ${\widehat{A}}^{\dag}\widehat{A}$, ${\widehat{B}}^{\dag}\widehat{B}$ and ${\widehat{a}}_{n}^{\dag}{\widehat{a}}_{n}$ (with $n\in \left\{1,2,3\right\}$), respectively. These fields exhibit independent fluctuation, and can only be conjugate pairs in the mean photon number. Parameter ${\eta }_{a}$ (with *a* $\in \left\{1\hspace{0.33em}to\hspace{0.33em}8\right\}$) refers to the Gaussian noise with correlation ${\eta }_{i}(z)=0$ and $\eta_{i} \left( z \right)\eta_{j} \left( {z^{\prime}} \right) = \delta_{ij} \delta \left( {z - z^{\prime}} \right)$. For the convenience of numerical simulation, the model parameters in Eq. (10) have been cast to the dimensionless forms using Ω and *J* for the mismatched frequency with the assumption that *J* = *J*_1_ = *J*_2_ = *J*_3_.

## Criteria for the nonclassicality

The time evolution Mandel *Q*_*m*_ parameter^37^ is a reliable way of classifying the statistical distribution property of mean fields. A positive *Q*_*m*_ denotes super-Poissonian statistics of light, whereas a negative *Q*_*m*_ indicates sub-Poissonian statistics of quantum phenomena with no classical analogy. In general, the Mandel *Q*_*m*_ parameter can be written as

11 $$ Q_{m} = \frac{{(\Delta \,\hat{n}^{2} )}}{{\hat{n}}} - 1, $$

where ($\Delta {\widehat{n}}^{2}$) refers to the variance of photon number $\widehat{n}={\widehat{A}}^{\dag}\widehat{A}$ governed by $(\Delta {\widehat{n}}^{2})=({\widehat{n}}^{2})-(\widehat{n}{)}^{2}$. Herein, we look at the normal-ordered property of mean-field at FH in *χ*^(2)^ waveguides. Considering the property of operator averages $\left\langle {\hat{n}} \right\rangle = \left\langle {\left| \varsigma \right|^{2} } \right\rangle_{P}$ and $\left\langle {\hat{A}^{\dag 2} \hat{A}^{2} } \right\rangle_{P} = \left\langle {\hat{n}^{2} } \right\rangle - \left\langle {\hat{n}} \right\rangle = \left\langle {\left| \varsigma \right|^{4} } \right\rangle_{P}$, the Mandel *Q*_*m*_ parameter can be written in the *P* representation form as

12 $$ Q_{m} = \frac{{\left\langle {\left| \varsigma \right|^{4} } \right\rangle_{P} - \left\langle {\left| \varsigma \right|^{2} } \right\rangle_{P}^{2} }}{{\left\langle {\left| \varsigma \right|^{2} } \right\rangle_{P} }}, $$

with 〈•〉_*P*_ being the classical average of *Q*_*m*_ trajectories concerning *P*$\left(\varsigma \right)$. Different metrics for detecting entanglement in the context of this study are available in the literature, for example, Cauchy-Schwarz^38^, Duan^39^, and Hillery-Zubairy^40^ criteria. In this paper, we use the Hillery-Zubairy criteria for bipartite inseparability to investigate the possibility of entanglement, because they are experimentally realizable and have a simple expression. In the present case, the modes of field can be written as^40^

13a $$ \varepsilon_{Aa} = \left\langle {\hat{A}^{\dag } \hat{A}\hat{a}^{\dag } \hat{a}} \right\rangle - \left| {\left\langle {\hat{A}\hat{a}^{\dag } } \right\rangle } \right|^{2} $$

13b $$ \varepsilon ^{\prime}_{Aa} = \left\langle {\hat{A}^{\dag } \hat{A}} \right\rangle \left\langle {\hat{a}^{\dag } \hat{b}} \right\rangle - \left| {\left\langle {\hat{A}\hat{a}} \right\rangle } \right|^{2} , $$

where the entanglement witness is positive if the entanglement correlation ${\varepsilon }_{Aa}$ or $\varepsilon {^{\prime}}_{Aa}$ is less than zero. To investigate squeezing, we define the single-mode field quadrature in the *χ*^(2)^ waveguides as

14 $$ \hat{X}_{1} = \frac{1}{2}\left[ {\hat{A} + \hat{A}^{\dag } } \right],\quad \hat{Y}_{1} = \frac{1}{2i}\left[ {\hat{A} - \hat{A}^{\dag } } \right]. $$

By substituting $\hat{A},\hat{A}^{ \dag } \Rightarrow \hat{a}_{n} ,\hat{a}_{n}^{ \dag} ,\;\;n \in \left\{ {1,2,3} \right\}$, the quadrature expression for surrounding fields can be obtained. In form of the stochastic field, the variance of Eq. (14) yields

15a $$ \left\langle {\left( {\Delta \hat{X}_{1} } \right)^{2} } \right\rangle = \frac{1}{4}\left\{ {\left\langle {\varsigma^{2} } \right\rangle + 2\left\langle {\varsigma^{*} \varsigma } \right\rangle + \left\langle {\varsigma^{*2} } \right\rangle + 1 - \left\langle \varsigma \right\rangle^{2} - 2\left\langle \varsigma \right\rangle \left\langle {\varsigma^{*} } \right\rangle - \left\langle {\varsigma^{*} } \right\rangle^{2} } \right\}, $$

15b $$ \left\langle {\left( {\Delta \hat{Y}_{1} } \right)^{2} } \right\rangle = \frac{1}{4}\left\{ { - \left\langle {\varsigma^{2} } \right\rangle + 2\left\langle {\varsigma^{*} \varsigma } \right\rangle - \left\langle {\varsigma^{*2} } \right\rangle + 1 + \left\langle \varsigma \right\rangle^{2} - 2\left\langle \varsigma \right\rangle \left\langle \varsigma \right\rangle^{*} + \left\langle {\varsigma^{*} } \right\rangle^{2} } \right\}. $$

Since we are also interested in mixed-mode squeezing, we have expanded Eq. (14) to account for the compound mode correlation as

16 $$ \hat{X}_{y} = \hat{X}_{1} + \frac{1}{2}\left[ {\sum\limits_{j = 1}^{n} {\left( {\hat{a}_{j} + \hat{a}_{j}^{\dag } } \right)} } \right],\quad \hat{Y}_{y} = \hat{Y}_{1} + \frac{1}{2i}\left[ {\sum\limits_{j = 1}^{n} {\left( {\hat{a}_{j} - \hat{a}_{j}^{\dag } } \right)} } \right]. $$

In Eq. (16), the mode of interaction is determined by the subscript *y,* (*y* = *n* + 1), i.e., *y* = 2 for two-mode, *y* = 3 for three-mode and *y* = 4 for four-mode. For *f* = 3, the maximal mixed-mode interaction is limited to *y* = 4 for the quad-channel system, and the variances of the field quadrature can be written as

17a $$ \begin{aligned} \left\langle {\left( {\Delta \hat{X}_{4} } \right)^{2} } \right\rangle & = \frac{\lambda }{2}\left[ {2\left\langle {\sum\limits_{n = 1}^{f} {\left\{ {\varsigma \alpha_{n} + \varsigma^{*} \alpha_{n} + \varsigma^{*} \alpha_{n}^{*} + \alpha_{n}^{*} \varsigma + \alpha_{1}^{*} \alpha_{n} + \alpha_{2}^{*} \alpha_{n} + \alpha_{3}^{*} \alpha_{n} } \right\}} } \right.} \right. \\ & \quad + \alpha_{1} \alpha_{2} + \alpha_{1} \alpha_{3} + \alpha_{2} \alpha_{3} + \alpha_{1}^{*} \alpha_{2}^{*} + \alpha_{1}^{*} \alpha_{3}^{*} + \alpha_{2}^{*} \alpha_{3}^{*} + \varsigma^{*} \varsigma \left. { + \lambda \left( {\varsigma \varsigma + \varsigma^{*} \varsigma^{*} + \sum\limits_{n = 1}^{f} {\left\{ {\alpha_{n} \alpha_{n} + \alpha_{n}^{*} \alpha_{n}^{*} + f} \right\}} } \right)} \right\rangle \\ & \quad - 2\left( {\lambda \left( {\left\langle \varsigma \right\rangle \left\langle \varsigma \right\rangle + \left\langle {\varsigma^{*} } \right\rangle \left\langle {\varsigma^{*} } \right\rangle } \right) + \sum\limits_{n = 1}^{f} {\left\{ {\left\langle {\alpha_{n} } \right\rangle \left\langle {\alpha_{n} } \right\rangle + \left\langle {\alpha_{n}^{*} } \right\rangle \left\langle {\alpha_{n}^{*} } \right\rangle } \right\}} } \right. + \sum\limits_{n = 2}^{f} {\left\{ {\left\langle \varsigma \right\rangle \left\langle {\alpha_{n} } \right\rangle + \left\langle {\varsigma^{*} } \right\rangle \left\langle {\alpha_{n} } \right\rangle + \left\langle {\varsigma^{*} } \right\rangle \left\langle {\varsigma^{*} } \right\rangle } \right.} \\ & \quad + \left\langle {\alpha_{n}^{*} } \right\rangle \left\langle \varsigma \right\rangle + \left\langle {\alpha_{1}^{*} } \right\rangle \left\langle {\alpha_{n} } \right\rangle \left. { + \left\langle {\alpha_{2}^{*} } \right\rangle \left\langle {\alpha_{n} } \right\rangle + \left\langle {\alpha_{3}^{*} } \right\rangle \left\langle {\alpha_{n} } \right\rangle } \right\} + \left\langle {\alpha_{1} } \right\rangle \left\langle {\alpha_{2} } \right\rangle + \left\langle {\alpha_{1} } \right\rangle \left\langle {\alpha_{3} } \right\rangle + \left\langle {\alpha_{2} } \right\rangle \left\langle {\alpha_{3} } \right\rangle \\ & \quad \left. {\left. { + \left\langle {\alpha_{1}^{*} } \right\rangle \left\langle {\alpha_{2}^{*} } \right\rangle + \left\langle {\alpha_{1}^{*} } \right\rangle \left\langle {\alpha_{3}^{*} } \right\rangle + \left\langle {\alpha_{2}^{*} } \right\rangle \left\langle {\alpha_{3}^{*} } \right\rangle + \left\langle {\varsigma^{*} } \right\rangle \left\langle \varsigma \right\rangle } \right\}} \right], \\ \end{aligned} $$

17b $$ \begin{aligned}   \left\langle {\left( {\Delta \hat{Y}_{4} } \right)^{2} } \right\rangle  &  = \frac{\lambda }{2}\left[ { - 2\left\langle {\sum\limits_{{n = 1}}^{f} {\left\{ {\varsigma \alpha _{n}  - \varsigma ^{*} \alpha _{n}  + \varsigma ^{*} \alpha _{n}^{*}  - \alpha _{n}^{*} \varsigma  - \alpha _{1}^{*} \alpha _{n}  - \alpha _{2}^{*} \alpha _{n}  - \alpha _{3}^{*} \alpha _{n} } \right\}}  + \alpha _{1} \alpha _{2}  + \alpha _{1} \alpha _{3}  + \alpha _{2} \alpha _{3} } \right.} \right. \\     & \quad \left. { + \alpha _{1}^{*} \alpha _{2}^{*}  + \alpha _{1}^{*} \alpha _{3}^{*}  + \alpha _{2}^{*} \alpha _{3}^{*}  - \varsigma ^{*} \varsigma  + \lambda \left( {\varsigma \varsigma  + \varsigma ^{*} \varsigma ^{*}  + \sum\limits_{{n = 1}}^{f} {\left\{ {\alpha _{n} \alpha _{n}  + \alpha _{n}^{*} \alpha _{n}^{*}  - f} \right\}} } \right)} \right\rangle  \\     & \quad  - 2\left( {\lambda \left( {\left\langle \varsigma  \right\rangle \left\langle \varsigma  \right\rangle  + \left\langle {\varsigma ^{*} } \right\rangle \left\langle {\varsigma ^{*} } \right\rangle \sum\limits_{{n = 1}}^{f} {\left\{ {\left\langle {\alpha _{n} } \right\rangle \left\langle {\alpha _{n} } \right\rangle  + \left\langle {\alpha _{n}^{*} } \right\rangle \left\langle {\alpha _{n}^{*} } \right\rangle } \right\}} } \right) + \sum\limits_{{n = 1}}^{f} {\left\{ {\left\langle \varsigma  \right\rangle \left\langle {\alpha _{n} } \right\rangle  - \left\langle {\varsigma ^{*} } \right\rangle \left\langle {\alpha _{n} } \right\rangle  + \left\langle {\varsigma ^{*} } \right\rangle \left\langle {\varsigma ^{*} } \right\rangle } \right.} } \right. \\     & \left. {\quad  - \left\langle {\alpha _{n}^{*} } \right\rangle \left\langle \varsigma  \right\rangle  - \left\langle {\alpha _{1}^{*} } \right\rangle \left\langle {\alpha _{n} } \right\rangle  - \left\langle {\alpha _{2}^{*} } \right\rangle \left\langle {\alpha _{n} } \right\rangle  - \left\langle {\alpha _{3}^{*} } \right\rangle \left\langle {\alpha _{n} } \right\rangle } \right\} + \left\langle {\alpha _{1} } \right\rangle \left\langle {\alpha _{2} } \right\rangle  + \left\langle {\alpha _{1} } \right\rangle \left\langle {\alpha _{3} } \right\rangle  + \left\langle {\alpha _{2} } \right\rangle \left\langle {\alpha _{3} } \right\rangle  \\     & \left. {\left. {\quad  + \left\langle {\alpha _{1}^{*} } \right\rangle \left\langle {\alpha _{2}^{*} } \right\rangle  + \left\langle {\alpha _{1}^{*} } \right\rangle \left\langle {\alpha _{3}^{*} } \right\rangle  + \left\langle {\alpha _{2}^{*} } \right\rangle \left\langle {\alpha _{3}^{*} } \right\rangle  + \left\langle {\varsigma ^{*} } \right\rangle \left\langle \varsigma  \right\rangle } \right\}} \right], \\  \end{aligned}  $$

where *λ* = 0.5. If any of the field quadrature variances fluctuate below the standard quantum limit, causing the temporal evolution to be less than 0, the system is producing squeezed light in the respective quadrature *S*_*X*_, *S*_*Y*_, in accordance with the expressions

18 $$ S_{X,1} = 4\langle (\Delta \hat{X}_{1} )^{2} \rangle - \left| 1 \right| \le 0,\quad S_{Y,1} = 4\langle (\Delta \hat{Y}_{1} )^{2} \rangle - \left| 1 \right| \le 0, $$

for single-mode and

19 $$ S_{X,y} = 4\langle (\Delta \hat{X}_{y} )^{2} \rangle - \left| y \right| \le 0,\;\;\;\;S_{Y,y} = 4\langle (\Delta \hat{Y}_{y} )^{2} \rangle - \left| y \right| \le 0, $$

for mixed-mode.

## The nonclassical features

### Sub-Poissonian photon number

To investigate the nonclassical features, Eq. (10) was solved over 10^4^ stochastic trajectories using the Runge–Kutta (RK4) method implementing different states of initialization, i.e., asymmetrical coherent (*χ*^(2)^)-vacuum (*χ*^(3)^, *χ*^(1)^), vacuum (*χ*^(2)^)-coherent (*χ*^(3)^, *χ*^(1)^) and symmetrical coherent (*χ*^(2)^)-coherent (*χ*^(3)^, *χ*^(1)^). In this sort of interaction, the input fields are commonly initialized with a combination of coherent states in all channels, or at least one channel is initialized with a coherent state while the rest is in a vacuum. For example, see^41^ and references therein. Therefore, in the present investigation, these initialization states are used to demonstrate various nonclassical aspects of the system. The nonclassical features are calculated, examined, and discussed throughout the study, however, only the initialization states where the features are most optimal are graphically shown.

**Figure 2:**
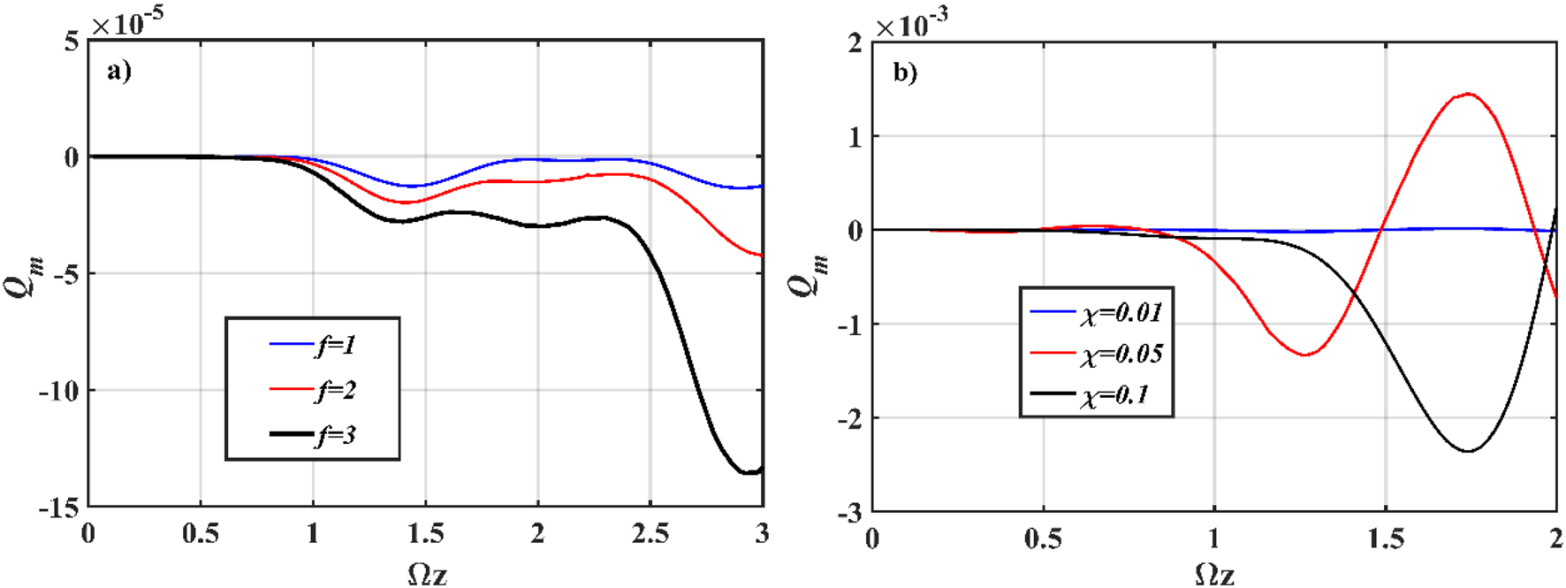
Numerical simulation plots of Eq. (12); evolution of *Q*_*m*_ parameter in *χ*^(2)^ waveguide for (**a**) different numbers of *χ*^(3)^ waveguides with *χ* = 0.01; (**b**) different values of *χ*. The other input parameters are fixed at* ς* = 2, *α*_n_ = 0 (asymmetrical coherent (*χ*^(2)^)-vacuum (*χ*^(3)^)), Ω = *ω* = 2, *J*_*n*_ = 2, *k*_*n*_ = 0, *g* = 10^–7^ for *n* = 1, 2, 3.

It is worth noting that the magnitude of the studied parameters ranges from the order of 10^–6^ to 10^–1^ as a result of the dimensionless interaction length and initialization state chosen. The magnitude is amplified in proportion to the interaction length and the value of the initialization state. In general, if the system is launched with symmetrical coherent initialization in all channels, the sub-Poissonian property of the FH field in the *χ*^(2)^ waveguide will be reduced. Alternatively, a more pronounced sub-Poissonian property is observed, if the input source is first launched from the *χ*^(3)^ waveguides for the asymmetric state of initialization. Figure 2 illustrates the property of sub-Poissonian photon in relation to (a) the number of *χ*^(3)^ waveguides, and (b) the strength of second-order nonlinearity *χ*^(2)^. The sub-Poissonian property is independent of the number of *χ*^(3)^ waveguides for symmetrical initialization states. However, as the number of *χ*^(3)^ waveguides with asymmetric initialization states increases, the sub-Poissonian property rises. At *χ* ≥ 0.1, the sub-Poissonian property is sufficiently stronger due to the strength of second-order nonlinearity *χ*^(2)^. However, as we can see in Eq. (10), the Gaussian noise is a function of *χ*. Increasing the value of *χ* in the *P* representation can affect the stability of integration. Therefore, in the present consideration, we limit the value of *χ* to 0.1.

**Figure 3:**
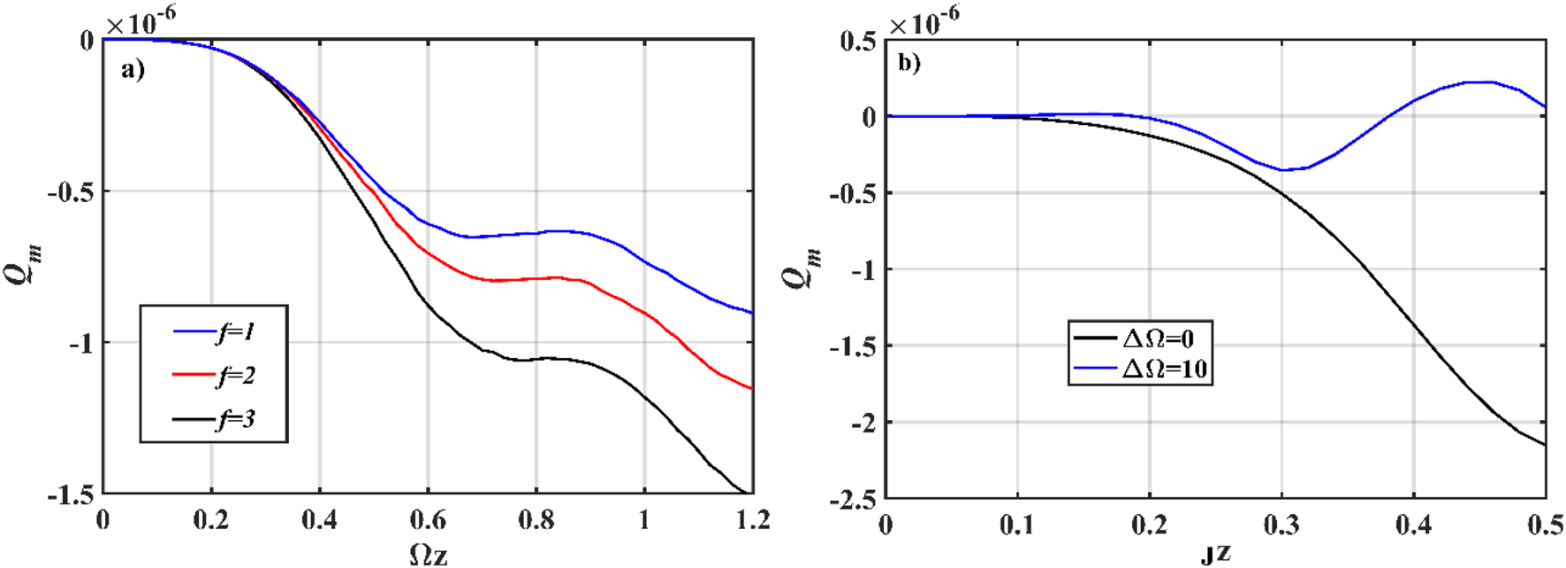
Numerical simulation plots of Eq. (12); evolution of *Q*_*m*_ parameter in *χ*^(2)^ waveguide for (**a**) different numbers of *χ*^(1)^ waveguides with *g* = 0, Ω = *ω* = 2; (**b**) different mismatched frequencies, *g* = 10^–7^ (*χ*^(2)^–*χ*^(3)^ type interaction). The other input parameters are fixed at* ς* = 2, *α*_*n*_ = 0 (asymmetrical coherent (*χ*^(2)^)-vacuum (*χ*^(3)^)), *J*_*n*_ = 2, *k*_*n*_ = 0, *χ* = 0.01 for *n* = 1, 2, 3.

To further investigate the effect of *χ*^(3)^ nonlinearity on the sub-Poissonian property of *χ*^(2)^ waveguide, we analyze the evolution of *Q*_*m*_ parameter for different values of *g*. In the surrounding waveguides, setting *g* = 0 eliminates the nonlinear effect, and in such conditions, it is known that *χ*^(1)^ is incapable of producing nonclassical effects. If these waveguides are allowed to evanescently coupled with other nonlinear waveguides, however, some important correlations can emerge (Fig. 3a). In contrast to Fig. 2a, the main remark for a *χ*^(2)^–*χ*^(1)^ type interaction is that removing the *χ*^(3)^ effect will reduce the sub-Poissonian property (of the photon) in *χ*^(2)^ waveguide. The maximum threshold for sub-Poissonian properties, on the other hand, rises in direct proportion to the number of *χ*^(1)^ waveguides. The evolution of *Q*_*m*_ parameter is recurring in the Poissonian and sub-Poissonian regions for asymmetric initialization states, regardless of where the light was launched in the beginning. On varying the value of *k*_*n*_ from 0 to 0.5*J*_*n*_, we observe that the trajectory is periodic along the vertical line at *Q*_*m*_ = 0, thereby suggesting it to be less sub-Poissonian. Furthermore, we introduce the influence of phase-mismatch on the property of photon by varying the operating frequency for Ω ≠ *ω*, where ∆Ω = Ω–*ω*, as shown in Fig. 3b. It appears that, in the absence of symmetry in the operating frequencies between the center and surrounding waveguides, the *Q*_*m*_ parameter corresponds to an increase in sub-Poissonian property, and peaked at ∆Ω = 10, for both *χ*^(2)^–*χ*^(3)^ and *χ*^(2)^–*χ*^(1)^ types of interaction.

### Quantum entanglement

**Figure 4:**
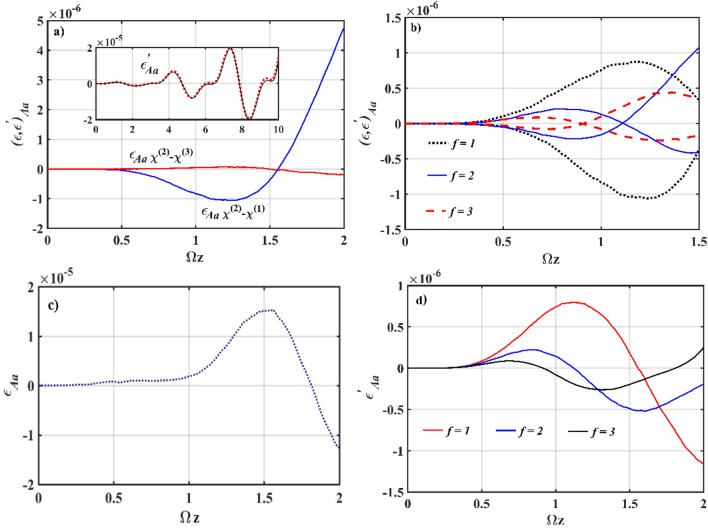
Numerical simulation plots of Eq. (13); (**a**) comparison between *ε*_*Aa*_ for *g* = 0 and *g* = 10^–7^ (inset: comparison between *ε*′_*Aa*_ for *g* = 0 (solid line) and *g* = 10^–7^ (dotted line)), (**b**) entanglement for different values of *f* at *g* = 10^–7^ (**c**) entanglement *ε*_*Aa*_ at *ς* = 0, *α*_*n*_ = 2, *g* = 10^–7^ and (**d**) entanglement for different values of *f* at *g* = 0. The other input parameters are fixed at *ς* = 2, *α*_*n*_ = 0 (asymmetrical coherent (*χ*^(2)^)-vacuum (*χ*^(3)^)), Ω = *ω* = 2, *J*_*n*_ = 2, *k*_*n*_ = 0, *g* = 10^–7^, *χ* = 0.01 for *n* = 1, 2, 3.

In Fig. 4a, prediction for the possibility of entanglement occurring between the central and surrounding waveguides is demonstrated for both the *χ*^(2)^–*χ*^(3)^ and *χ*^(2)^–*χ*^(1)^ interactions. Numerical estimates of the entanglement criteria for both cases show that the entanglement in the *ε*′_*Aa*_ component is very similar (inset of Fig. 4a). On the other component *ε*_*Aa*_, the *χ*^(2)^–*χ*^(1)^ interaction shows a stronger entanglement. However, unlike the *ε*′_*Aa*_ component, where the intensity was determined by Ωz, *ε*_*Aa*_ entangled only for short evolution, with the sign for entanglement disappearing around Ωz ≈ 1.5. In the presence of *χ*^(3)^ nonlinearity (Fig. 4b), entanglement is optimized when the system is running at *f* = 1, with the maximal entanglement appearing at larger Ωz. We find that increasing the *χ*^(3)^ waveguides primarily reduces entanglement, and the behavior of having a stronger intensity in later evolution vanishes. The entanglement is minimal at *f* = 3; nevertheless, it can be amplified if suitable interaction parameters are selected.

To generate maximal entanglement, the initialization state must be carefully chosen. When the *χ*^(2)^ waveguide is asymmetrically initialized with a coherent state, while the *χ*^(3)^ waveguides are in a vacuum, the strongest sign of entanglement between the *χ*^(2)^ and *χ*^(3)^ waveguides is observed, which is most visible in the *ε*′_*Aa*_ component. In the early evolution of *ε*_*Aa*_, a similar sign of entanglement may appear if the state of initialization is reversed, i.e., the input is launched in the *χ*^(3)^ waveguides first. Unlike the previous choice of initialization, however, the tendency for *ε* to increase in magnitude as Ωz increases fades away (Fig. 4c). Furthermore, the amount of entanglement is reduced in the symmetrical initialization state. However, compared to the case when the *χ*^(2)^ waveguide is prepared in a vacuum, the amount of entanglement can be of high intensity, particularly in the *ε*′_*Aa*_ component.

We observe that the maximal entanglement is independent of the asymmetrical initialization condition in the absence of *χ*^(3)^ nonlinearity (Fig. 4d). Also, the strength of entanglement is similar in both the cases of initialization, and we notice the same behavior of generating stronger entanglement at larger Ωz. This is not the case in the symmetrical initialization state, where the strongest entanglement appears at early evolution for a short interaction length. Entanglement decreases with the increase of *χ*^(1)^ waveguides, similar to non-zero *g*; however, if the *χ*^(1)^ waveguides interact so that *k*_*n*_ is non-zero, the amount of entanglement increases, relative to *f* = 3 with *k*_*n*_ = 0. Furthermore, it makes no difference whether all the *χ*^(1)^ waveguides are connected or not; as long as one of the coupling constants is non-zero, a comparable amount of entanglement is observed.

### Squeezing

**Figure 5:**
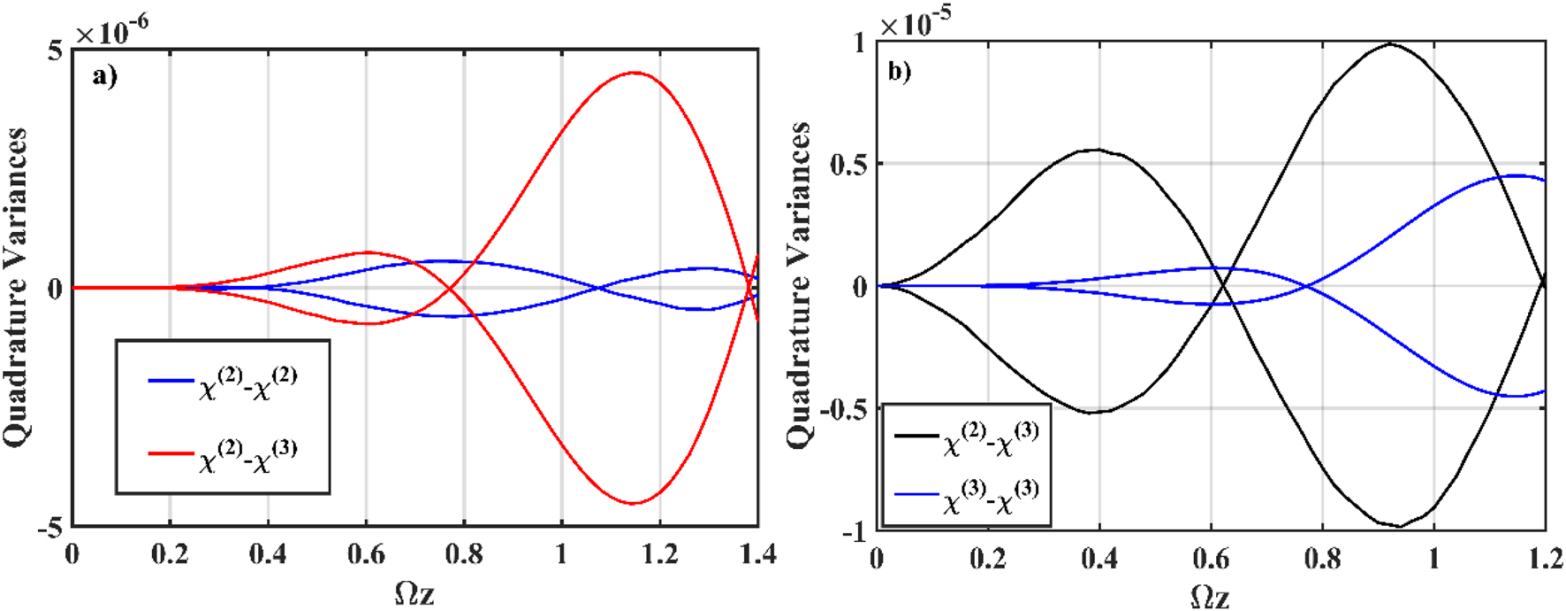
Numerical simulation plots of Eq. (18) *S*_*X*,1_, *S*_*Y*,1_; (**a**) *χ*^(2)^–*χ*^(3)^ versus QOD; (**b**) *χ*^(2)^–*χ*^(3)^ versus KNC. The other input parameters are fixed as* ς* = 2, *α*_1_ = *α*_2_ = *α*_3_ = 0, Ω = *ω* = 2, *J*_*n*_ = 2, *k*_*n*_ = 0, *g* = 10^–7^ and *χ* = 0.01.

Figure 5 depicts the squeezing that occurs in the *χ*^(2)^ waveguide as a result of the addition of asymmetric nonlinearity. To address the significance of these results in terms of the addition of *χ*^(3)^, the findings are contrasted with the results obtained from the symmetrical *χ*^(2)^–*χ*^(2)^ interaction, i.e., the Quantum Optical Dimer (QOD) discussed by Mallon et al.^42^ (Fig. 5a), and the symmetrical Kerr Nonlinear Coupler (KNC) *χ*^(3)^–*χ*^(3)^ by Ibrahim et al.^43^ (Fig. 5b), using the same input parameters. In Fig. 5a, introducing *χ*^(2)^–*χ*^(3)^ interaction as an alternative to QOD increases the strength of squeezing. We observe that squeezing seems to occur at various phases at first. Nonetheless, around Ωz ≈ 1.5, the phase at which squeezing appears in both cases is the same.

The maximal squeezing is most often seen in the waveguide into which the light is first launched. On the contrary, we find that the vacuum *χ*^(3)^ waveguides exhibit the most squeezing. Similarly, if the *χ*^(3)^ waveguide is asymmetrically initialized with a coherent state, maximal squeezing occurs in the *χ*^(3)^ waveguide. Under this condition, stronger squeezing is expected in the *χ*^(2)^ waveguide as well. Figure 5b shows a comparison of squeezing in the present system versus KNC. Squeezing will increase if one of the *χ*^(3)^ waveguides in the KNC is replaced with the *χ*^(2)^ waveguide. This only occurs in the *χ*^(2)^ waveguide at short evolution distances, as the quadrature evolution becomes more in step after Ωz > 2. However, stronger squeezing occurs in the *χ*^(3)^ waveguides.

**Figure 6:**
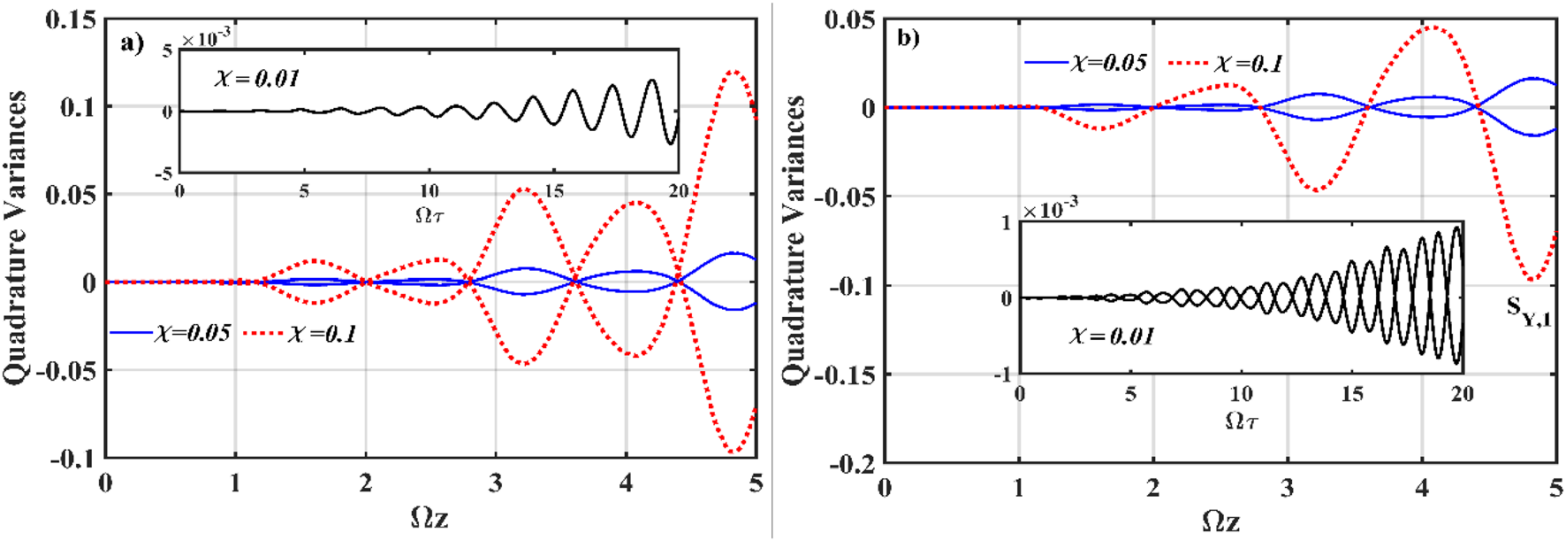
Numerical simulation plots of Eq. (18) *S*_*X*,1_, *S*_*Y*,1_ for different value of *χ*; (**a**) evolution of the quadrature variances in the *χ*^(2)^ waveguide (inset *S*_*X*,1_), and (**b**) evolution of the quadrature variances in the *χ*^(3)^ waveguide. The other input parameters are fixed as* ς* = 2, *α*_1_ = *α*_2_ = *α*_3_ = 0, Ω = *ω* = 2, *J*_*n*_ = 2, *k*_*n*_ = 0, *g* = 10^–7^ and *χ* = 0.01.

To illustrate the effect of *χ* on the quadrature evolution, Fig. 6 depicts the numerical simulation plots of Eq. (18) for various values of *χ* for both the *χ*^(2)^ and *χ*^(3)^ waveguides. Squeezing in FH of the *χ*^(2)^ waveguide with nonlinearity *χ* = 0.01 is shown in the inset of Fig. 6a for comparison with the squeezing produced at higher *χ*, such as 0.05 and 0.1. We notice that the system produces squeezing more efficiently when the input field is launched at a higher value of *χ*, resulting in rapid amplification in the magnitude of quadrature evolution below the standard quantum limit. Besides, at fixed *g*, such as *g* = 10^–7^, increasing *χ* appears to enhance squeezing in the *χ*^(3)^ waveguides as well. Kerr squeezing can be stronger at *χ* = 0.1 than at *χ* = 0.05 in the *χ*^(2)^ waveguide. However, at the same value of *χ*, such as *χ* = 0.1, the effect of amplification is most pronounced in the FH of the *χ*^(2)^ waveguide (Fig. 6b). Although only the *S*_*Y*_ component of Kerr squeezing is seen at *χ* = 0.1, the squeezing and anti-squeezing produced in the other component *S*_*X*_ are very similar.

**Figure 7:**
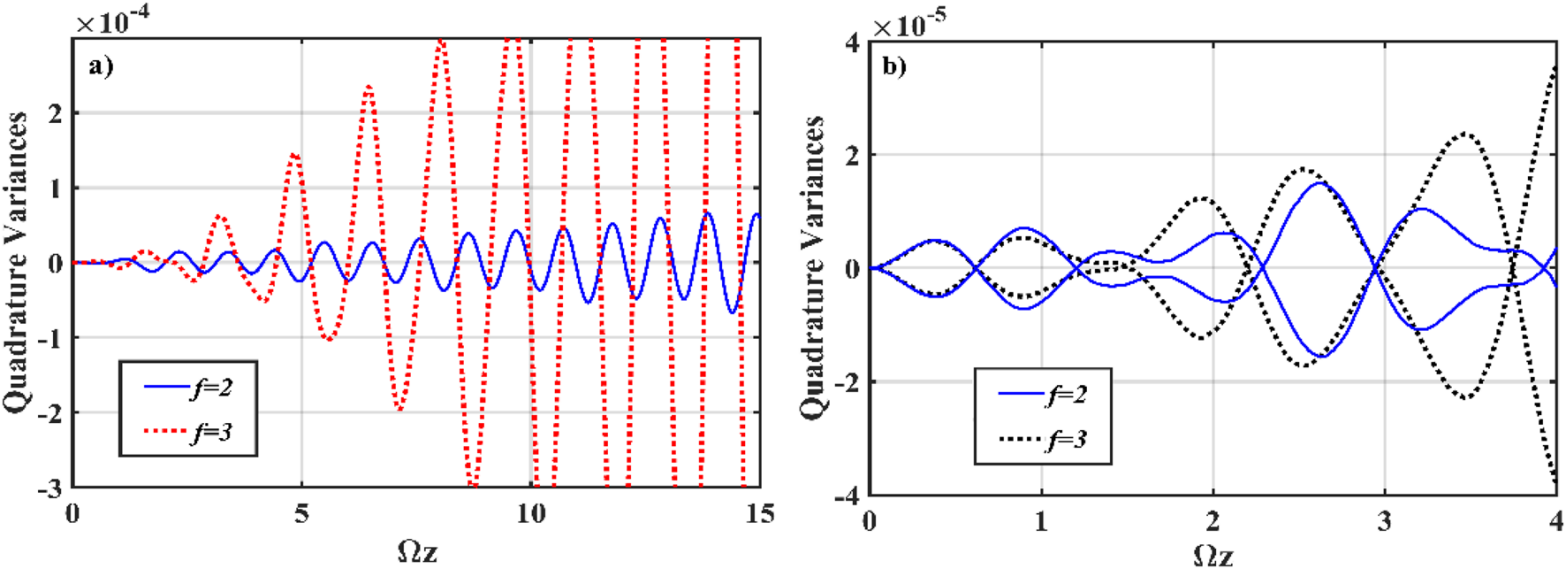
Numerical simulation plots of Eq. (18) for *χ*^(2)^–*χ*^(3)^ interaction with different numbers of *χ*^(3)^ waveguides; evolution of quadrature variances in the (**a**) *χ*^(2)^ waveguide (*S*_*X*,1_), and (**b**) *χ*^(3)^ waveguides (*S*_*X*,1_, *S*_*Y*,1_). The other input parameters are fixed at* ς* = 2, *α*_1_ = *α*_2_ = *α*_3_ = 0, Ω = *ω* = 2, *J*_*n*_ = 2, *k*_*n*_ = 0, *g* = 10^–7^ and *χ* = 0.01.

In Fig. 7, we display the generation of squeezing in both the *χ*^(2)^ (Fig. 7a) and *χ*^(3)^ (Fig. 7b) waveguides in relation to the coupling profiles and the number of channel waveguides involved in the interaction. Remarkably, by suitable manipulation of the coupling profiles between the interacting waveguides, it is possible to generate interesting nonclassical states. At *f* = 3, quantum correlations are efficiently produced on a multichannel basis, allowing for enhanced squeezing in all channels where the quadrature evolution oscillates periodically between a maximal squeezing and a standard quantum limit with the same oscillatory period. However, for this to occur, all *χ*^(3)^ waveguides must be kept isolated from one another by setting *k*_*n*_ = 0. Squeezing in all channels would be reduced if the simultaneous coupling between the *χ*^(3)^ waveguides is permitted, i.e., non-zero *k*_*n*_. Additionally, if all waveguides are equally pumped, at* ς* = *α*_*n*_ = 2 (not shown in the figure), squeezing produced in the *χ*^(2)^ and *χ*^(3)^ waveguides resemble Fig. 7a. Squeezing builds up smoothly in all waveguides for both the symmetric and asymmetric initialization states, and oscillatory maximal squeezing increases as Ωz increases.

**Figure 8:**
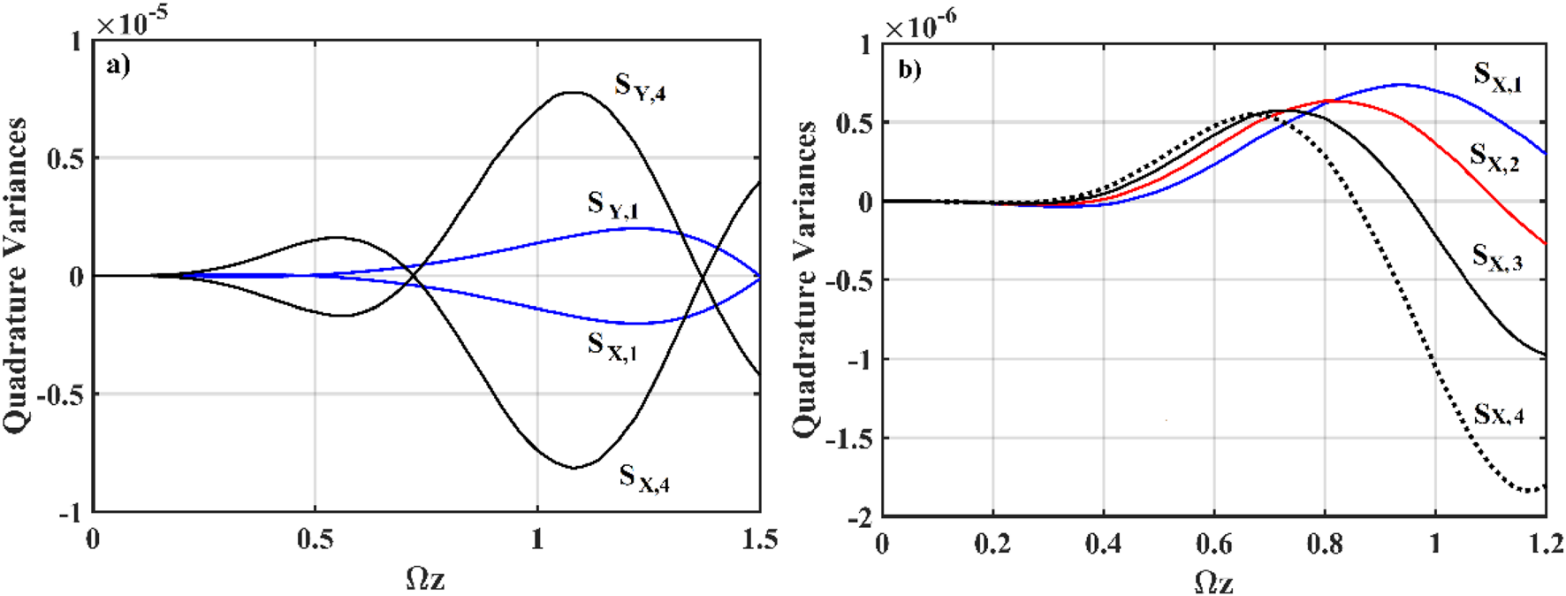
Numerical simulation plots of Eq. (19); (**a**) mixed-mode squeezing in the *χ*^(2)^ waveguide for *g* = 10^–7^ and (**b**) mixed-mode squeezing in the *χ*^(2)^ waveguide for *g* = 0. The other input parameters are fixed at *ς* = 2, *α*_1_ = *α*_2_ = *α*_3_ = 0, Ω = *ω* = 2, *J*_*n*_ = 2, *k*_*n*_ = 0 and *χ* = 0.01.

In addition to the increased number of *χ*^(3)^ waveguides, we further investigate the features obtained for mixed-mode correlations. Due to the multichannel interaction, this system is a natural medium for producing mixed-mode squeezing. For both the *χ*^(2)^–*χ*^(3)^ and *χ*^(2)^–*χ*^(1)^ types of interaction, Fig. 8 shows the results in terms of mixed-mode quadrature obtained from the numerical simulation of Eq. (19). Figure 8a depicts the evolution of mixed-mode squeezing versus single-mode for the *χ*^(2)^–*χ*^(3)^ interaction. We see that, on a mixed-mode basis, the squeezed quadrature outperforms the single-mode squeezing. Figure 8b depicts the *S*_*X*_ quadrature evolution of mixed-mode squeezing for the *χ*^(2)^–*χ*^(1)^ interaction, which demonstrates the behavior similar to that in Fig. 8a. Overall, adding multichannel interaction leads to the possibility of quantum correlation between the input fields, thereby implying that enhanced squeezing in the mixed-mode basis is to be predicted. Adding a different order of nonlinear interaction, such as *χ*^(2)^–*χ*^(3)^ and *χ*^(2)^–*χ*^(1)^, can boost squeezing even more.

**Figure 9:**
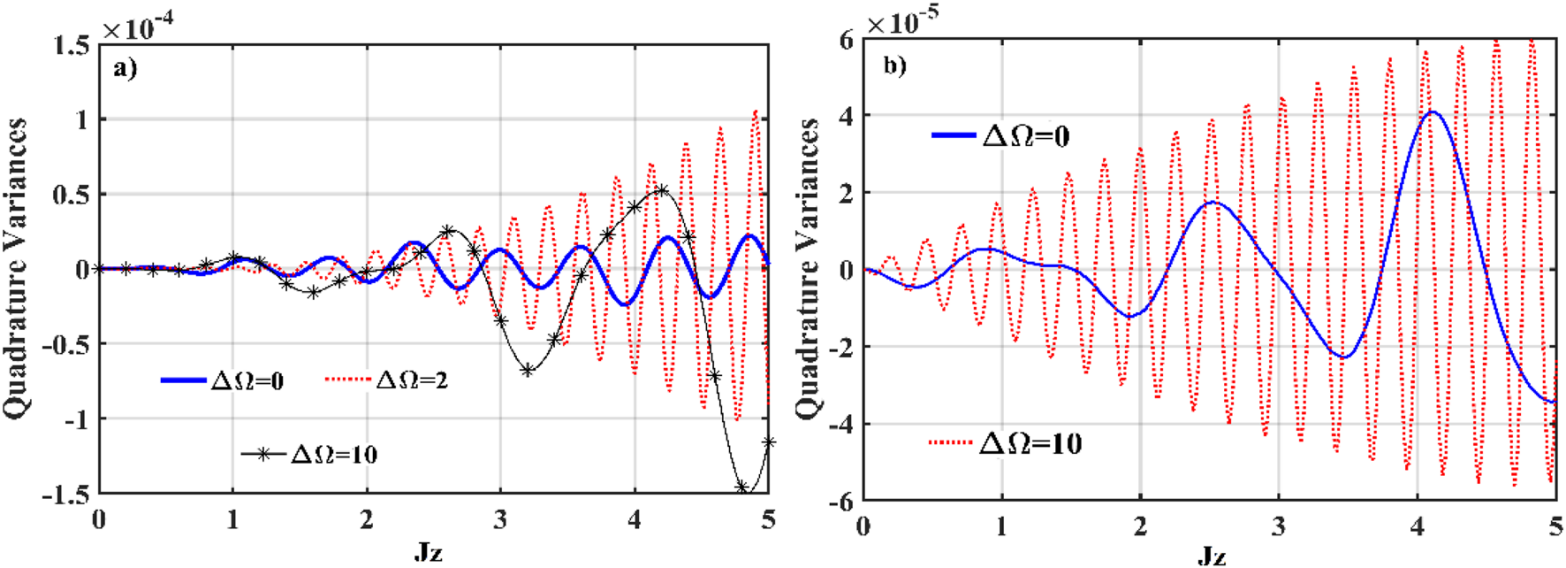
Numerical simulation plots of Eq. (18) with mismatched frequencies; (evolution of the quadrature variances (*S*_*X*,1_) in the (**a**) *χ*^(2)^ waveguide, and (**b**) *χ*^(3)^ waveguide. The other input prameters are fixed at *ς* = 2, *α*_1_ = *α*_2_ = *α*_3_ = 0, Ω–*ω* = ∆Ω = 0, 2, 10, *J*_*n*_ = 2, *k*_*n*_ = 0, *g* = 10^–7^ and *χ* = 0.01.

Figure 9 shows numerical simulation plots of Eq. (18) for various frequencies of the input modes, which can be used to determine squeezing with frequency mismatch. Given the frequency mismatch between the interacting modes in different waveguides for the *χ*^(2)^–*χ*^(3)^ interaction, we concentrate on the mismatched configurations: (a) ∆Ω = 0, (b) ∆Ω = 2, and (c) ∆Ω = 10, where ∆Ω is given by Ω–*ω*. Figure 9a shows the evolution of squeezed quadrature in the *χ*^(2)^ waveguide at various mismatched frequencies, with the least amount of squeezing expected at Ω = *ω*. When a mismatched frequency is present, the squeezed quadrature generally increases. For example, at ∆Ω = 2, the squeezed quadrature has a shorter oscillatory duration and exhibits more oscillations. Increasing the mismatch frequency further lengthens the oscillatory duration, resulting in a better prediction of squeezing at ∆Ω = 10. Figure 9b exhibits squeezing in the *S*_*X*_ quadrature as it evolves in the *χ*^(3)^ waveguides. Similarly, in the *χ*^(3)^ waveguides, ∆Ω = 10 induces maximal oscillatory squeezing, which increases as Ωz increases. In comparison to the maximal squeezed quadrature observed in the *χ*^(3)^ waveguides, mismatched frequency ∆Ω = 10 decreases the oscillatory time as squeezing is increased.

## Conclusion

The aforementioned investigation focuses on the numerical estimation of nonclassical properties of multichannel waveguides with the second and third-order nonlinear effects. It has been found that the initialization state must be carefully selected to achieve optimization. With an asymmetric initialization state, the sub-Poissonian property increases as the number of *χ*^(3)^ waveguides increases; it becomes sufficiently strong as the strength of *χ*^(2)^ approaches 0.1. Removing the effect of *χ*^(3)^ reduces the property in *χ*^(2)^ waveguides. However, the overall threshold for sub-Poissonian properties rises in direct proportion to the number of *χ*^(1)^ waveguides, and the *Q*_*m*_ parameter corresponds to an increase in the sub-Poissonian property when phase mismatched is present. Furthermore, entanglement is optimized when the system is operating at *f* = 1, and increasing the *χ*^(3)^ waveguides primarily reduces entanglement. The maximal entanglement is independent of the asymmetrical initialization condition in the absence of *χ*^(3)^ nonlinearity. and entanglement decreases with the increase of *χ*^(1)^ waveguides. Incorporating the *χ*^(2)^–*χ*^(3)^ interaction in the present system as an alternative to QOD and KNC increases the strength of squeezing, and a more effective squeezing can be achieved in all waveguides if the input field is launched at a higher value of *χ*. When multichannel interaction is added, a quantum correlation between the input fields is possible. That is, increased squeezing in the mixed-mode basis can be anticipated. Squeezing can be boosted even further by using a certain order of nonlinear interaction, such as *χ*^(2)^–*χ*^(3)^ and *χ*^(2)^–*χ*^(1)^. Finally, when a mismatched frequency is present, the squeezed quadrature generally increases, which increases as Ωz increases.

Overall, with more effective squeezing achieved in all channel waveguides, the present system with *χ*^(2)^–*χ*^(3)^ interaction can be a more efficient alternative to other versions of nonlinear couplers such as the quantum optical dimer (QOD) and the Kerr nonlinear coupler (KNC). Furthermore, the present system offers more flexibility in coupled-mode interactions in the form of possibilities of correlation between the modes in different waveguides. This provides a better mechanism for the generation of enhanced nonclassical effects.

## Data Availability

A MATLAB code was developed to generate these theoretical results. The code is available from the corresponding author on reasonable request.
